# *Descolea quercina* (Bolbitiaceae), a new species from moist temperate forests in Pakistan

**DOI:** 10.3897/mycokeys.27.14730

**Published:** 2017-11-09

**Authors:** Junaid Khan, Hassan Sher, Arooj Naseer, Abdul Nasir Khalid

**Affiliations:** 1 Center for Plant Sciences and Biodiversity, University of Swat, Swat, Pakistan; 2 Center for Undergraduate studies, University of the Punjab, Lahore, Pakistan; 3 Department of Botany, University of the Punjab, Lahore, Pakistan

**Keywords:** Basidiomycota, ectomycorrhiza, taxonomy

## Abstract

A new species, *Descolea quercina*, is described and illustrated from Northern parts of Khyber Pakhtunkhwa, Pakistan. It is characterized by medium to large basidiomata, squamose to squamose-granulose hygrophanous pileus, and limoniform, verrucose basidiospores with partly concrescent verrucae. Phylogenetic analyses of nuc rDNA region encompassing the internal transcribed spacers 1 and 2 along with 5.8S rDNA (ITS) and nuc 28S rDNA D1-D2 domains (28S) also confirmed it as a new species. A comparison with similar taxa is provided.

## Introduction

The genus *Descolea* Singer was based on *D. antarctica* Singer, which has agaricoid basidiomata with an annulus, thus resembling *Rozites* or *Pholiotina* spp. (Horak 1971). *Descolea* is currently placed in the *Bolbitiaceae* (Kirk et al. 2008) and is characterized by dry to viscid pileus with or without squamules, central stipe with striated annulus, ochraceous spore deposit, amygdaliform to limoniform, verrucose basidiospores with a smooth apiculus, and a hymeniform pileipellis (Horak 1971). *Descolea* was once considered to be restricted to the southern hemisphere, however, the known 15 species (Sharma and Kumar 2011) have a wide geographical distribution (Australia, India, Japan, Korea, New Guinae, New Zealand, Pakistan, Siberia, South America) (Horak 1971; Bougher and Malajczuk 1985; Niazi et al. 2007). From Pakistan, only *D. flavoannulata* (Lj.N. Vassiljeva) E. Horak was reported to date. During our macrofungal surveys, we collected a rare and interesting species of *Descolea* from two locations in Northern areas of Khyber Pakhtunkhwa, Pakistan. The species appeared unique and based on discrete morphological characteristics and sequences derived from nuc rDNA region encompassing the internal transcribed spacers 1 and 2 along with 5.8S rDNA (ITS) and nuc 28S rDNA D1-D2 domains (28S), it is described here as new to science.

## Materials and methods

### Collection and morphological characterization

Collections were made on routine mycological visits to the moist temperate *Quercus* dominated mixed forests of Malam Jabba (Swat district) and Toa valley (Shangla district), Khyber Pakhtunkhwa province, Pakistan. Basidiomata were collected following Lodge et al. (2004) and photographed in their natural habitats. Descriptions of the macro-characters are based on fresh collections and colored photographs. Color codes follow Munsell soil color charts (1975) and are presented in parenthesis after common color names.

Microscopic characters are based on free hand sections from fresh and dried specimens mounted in 5% (w/v) aqueous Potassium Hydroxide (KOH) solution. Measurements of anatomical structures are based on calibrated computer based software “PIXIMÈTRE version 5.9” connected to a compound microscope (BOECO, Model: BM120) and visualized through a microscopic camera (MVV 3000). A total of twenty basidiospores, basidia, cystidia and hyphae were measured from all the collections. For measurements; Q is the range of length/width (L/W) ratio of the total measured basidiospores; Qe is the average L/W ratio of all the measured basidiospores; Me is the average L × W of all the measured basidiospores. Surface of the basidiospores was studied both in 5% KOH solution and scanning electron microscopy (SEM).

### DNA extraction

DNA from herbarium specimens was extracted following the procedure mentioned in Peintner et al. (2001). A primer pair ITS1F (Gardes and Bruns 1993) and ITS4 (White et al. 1990) was used to amplify the ITS region and primer pair LR5 and LR0R (Vilgaly’s lab http://sites.biology.duke.edu/fungi/mycolab/primers.htm) was used to amplify the 28S region. Polymerase chain reactions (PCR) were performed in 25 µL volume per reaction. PCR procedure for ITS region consisted of initial 4 minutes denaturation at 94°C, 40 cycles of 1 minute at 94°C, 1 min at 55°C, 1 min at 72°C, and a final extension of 10 minutes at 72°C. PCR procedure for 28S region consisted of initial denaturation at 94°C for 2 minutes, 35 cycles of 94°C for 1 minute, 52°C for 1 minute, 72°C for 1 minute, and final extension at 72°C for 7 minutes. Visualization of PCR products were accomplished using 1% agarose gel added with 3 µL ethidium bromide and a UV illuminator. Sequencing of the amplified products was accomplished through outsourcing (BGI, Beijing Genomic Institute, Hong Kong).

### Phylogenetic analyses

The ITS region of the voucher collections MJ-1590, MJ-1590a and AST33 yielded a 725, 732 and 735 bp fragments respectively. Sequences of all the three specimens were used as a reference to BLAST against GenBank. All the query sequences matched 88% with *Descolea phlebophora* E. Horak (HQ533035) and *D. recedens* (Sacc.) Singer (KU523938) from New Zealand. Sequences of other genera, *Descomyces* Bougher & Castellano, *Timgrovea* Bougher & Castellano and *Setchelliogaster* Pouzar, were also downloaded for high similarity with query sequences and used in the subsequent phylogenetic analyses. *Hebeloma fastibile* (Pers.) P. Kumm (AF325643) and *H. circinans* (Quél.) Sacc. (JF908041) were selected as outgroup taxa (Peintner et al. 2001).

The 28S region yielded a 958 bp fragment for MJ-1590 and AST33, while the third collection (MJ-1590a) yielded a noisy sequence which was not included in the final analyses. The query sequences on blast showed 99% similarity with *Descolea recedens* (Sacc.) Singer (HQ827174), *Descolea maculata* Bougher (DQ457664) and *Descolea gunnii* (Berk. ex Massee) E. Horak (AF261523) from USA. Based on high similarity with query sequences, some unknown *Descomyces* species were also included in the phylogenetic analyses. *Hebeloma fastibile* (AY033139) and *H. affine* Smith, Evenson & Mitchel (FJ436324) were used as outgroup taxa.

DNA Sequences were aligned using online webPRANK tool at http://www.ebi.ac.uk/goldman-srv/webprank/ (Löytynoja and Goldman 2010). Maximum likelihood analyses for individual gene regions were performed via CIPRES Science Gateway (Miller et al. 2010) employing RAxML-HPC v.8. Rapid bootstrap analysis/search for best-scoring ML tree was configured for each dataset. For the bootstrapping phase, the GTRCAT model was selected. One thousand rapid bootstrap replicates were run. A bootstrap proportion of ≥ 70% was considered significant. Maximum parsimony (MP) analyses were performed using PAUP* 4.0b (Swofford 2002), with all characters of type unordered and equally weighted. Gaps were treated as missing data. Heuristic searches were performed with 1000 replicates with random taxon addition. MAXTREES was set to 5000 with MulTrees option in effect and TBR branch swapping. All characters were of type ‘unord’ and equally weighted.

## Results

### Molecular phylogenetic analyses

**Figure 1.**
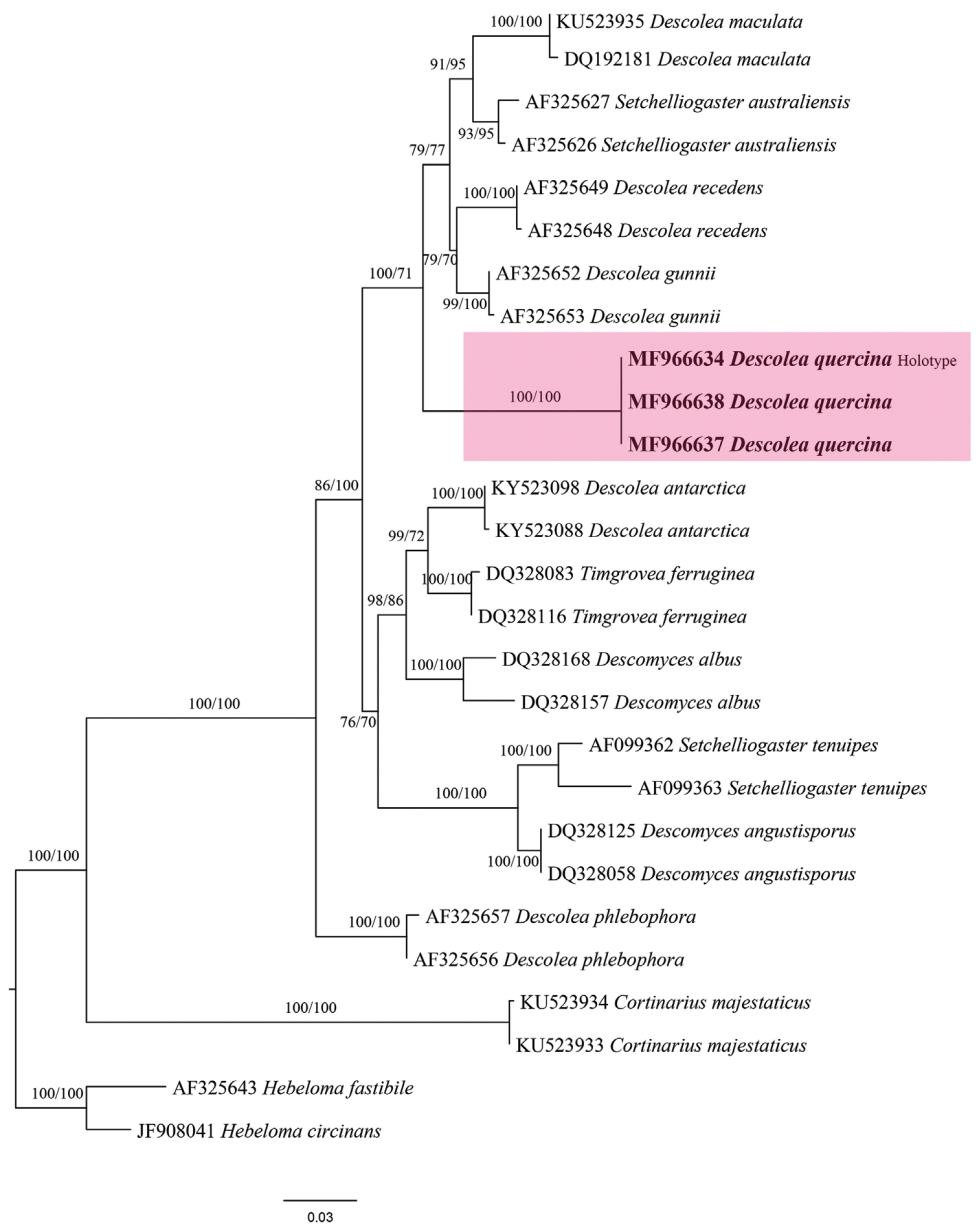
Phylogenetic relationship of *Descolaea quercina* and associated taxa inferred from ITS data. All positions with less than 70% site coverage were eliminated. Maximum likelihood and Maximum parsimony Bootstraps are shown close to the nodes. *Descolea quercina* is represented in boldface

The ITS based analysis involved 27 nucleotide sequences. There were a total of 694 characters in the alignment file of which 345 characters were constant, 45 variable characters were parsimony-uninformative while 304 variable characters were parsimony-informative. The tree resulting from the ITS based ML analysis (Fig. 1) was similar to the MP. The distribution of *Descolea* species among different clades is in conformity with Peintner et al. (2001). The sequences from the Pakistani collections (MJ-1590, MJ-1590a and AST33) formed a separate clade with robust bootstrap support (ML 100% and MP 71%), supporting its independent position.

**Figure 2.**
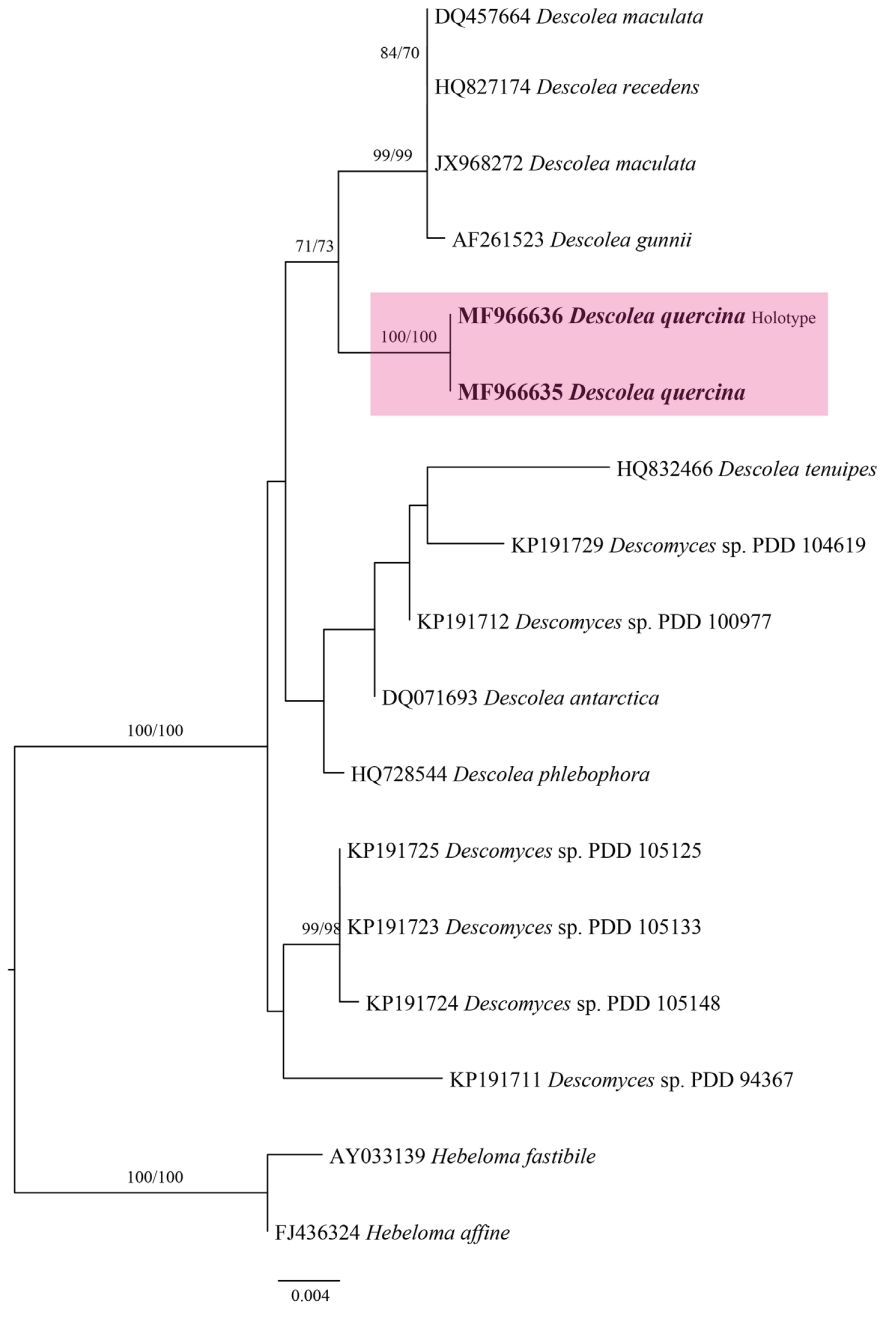
Phylogenetic relationship of *Descolaea quercina* and associated taxa inferred from 28S data. All positions with less than 70% site coverage were eliminated. Maximum likelihood and Maximum parsimony Bootstraps are shown by the nodes. *Descolea quercina* is represented in boldface.

The 28S based analysis involved 17 nucleotide sequences with a total of 941 characters, out of which 867 characters were constant, 16 variable characters were parsimony-uninformative and 58 variable characters were parsimony-informative. The ML phylogram (Fig. 2) was found congruent with MP phylogram (not shown). The sequences from Pakistani collections (MJ-1590 and AST33) formed a separate clade (Fig. 2), with was poorly supported by bootstrap values (ML 71% and MP 73 %), but tree topologies further support its unique position.

## Taxonomy

### Descolea quercina

Taxon classificationFungiAgaricalesBolbitiaceae

J. Khan & A. Naseer sp. nov.

820545

Figures 3 , 4 , 5

#### Type.

PAKISTAN. Khyber Pakhtunkhwa Province, Swat district, Malam Jabba valley, 1950 m alt., 25 July 2015, Junaid Khan, MJ-1590, (holotype: SWAT000135).

#### Diagnosis.

Basidiomata medium to large, pileus convex to convex-campanulate with a broad umbo in young stages, light yellowish brown to deep yellowish brown, surface dry, hygrophanous, squamose to squamose-granulose with striate margin; basidiospores limoniform, coarsely verrucose with partly concrescent verrucae.

**Figure 3.**
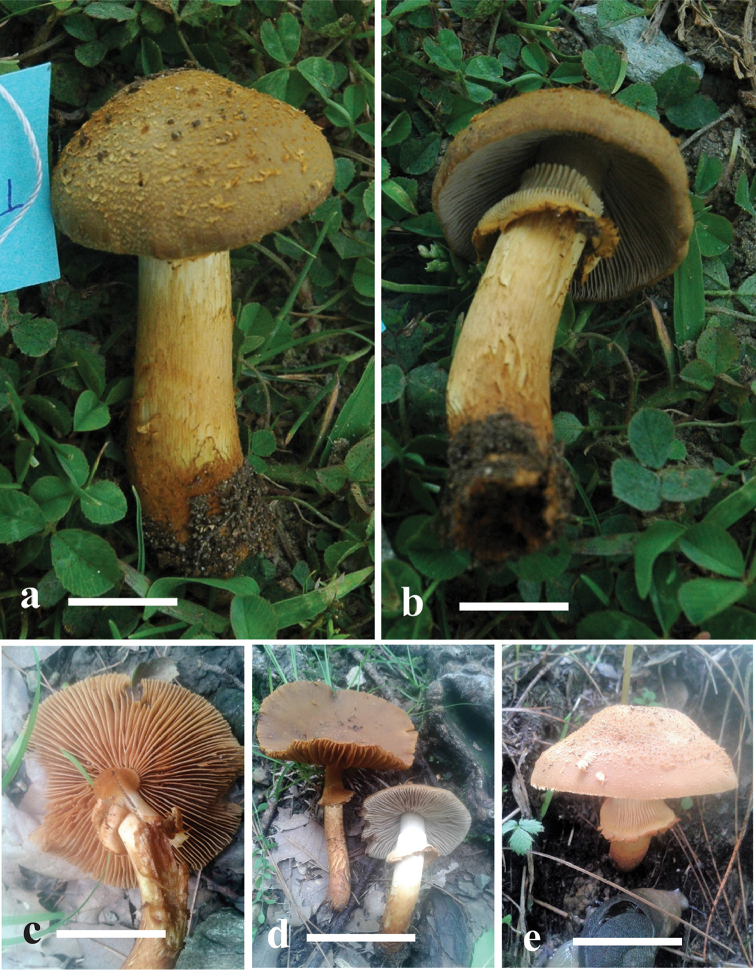
**a–e** Basidiomata of *Descolea quercina* sp.nov. **a, b** AST33 **c, d** MJ-1590 (Holotype) **e** Natural habitat (MJ-1590a). Scale bars 12mm for **a, b**; 40 mm for **c–e**

#### Description.

***Pileus*** 50–70 mm diameter, convex to convex-campanulate with a broad umbo when young, plane to plano-concave by maturity, light yellowish brown (7.5YR 7/4) to deep yellowish brown (10YR 3/8) with or without olivaceous tinge, surface hygrophanous, squamose to squamose-granulose, scales more or less concentrically arranged, loose, disappearing in age, margin striate; context strong yellowish brown (10YR 5/8), moist, thicker at the center (2–3 mm), color unchanging upon cutting. ***Lamellae*** adnexed, close, light grayish brown (7.5YR 6/4) in young specimens, yellowish brown in mature specimens (7.5YR 7/4), lamellar edge even, lamellulae present, mostly 3 in number, rarely single, often crisped at terminals, some lamellae forking near the stipe. ***Stipe*** 50–70 × 8–12 mm, central, thickening towards base, light yellowish brown (7.5 YR 7/4) to strong yellowish brown (10YR 4/8) and smooth above the ring , yellowish brown (10YR 5/6) and longitudinally fibrillose below the annulus; annulus membranous, concolorous with the lamellae, strongly striate on the upper surface, smooth to slightly scaly below, margin appendiculate; context fibrous, interior hollow at the center, flesh whitish above the annulus, yellowish brown (10YR 5/6) below, moist. Smell and taste rancid when cut.

***Basidiospores*** (10–) 11.5–13 (–14) × (6.5–) 6.7–8.6 (–9) µm, Q = 1.4–1.7 (–1.9), Me = 12.0 × 7.9 µm, Qe = 1.5, limoniform, with prominent papilla, coarsely verrucose, verrucae partly concrescent, with prominent smooth apiculus, perispore distinct, without germ-pore, plage smooth, rust brown in KOH. ***Basidia*** 25–40 × 8–12 µm, clavate, tetra-sterigmated, rarely bi sterigmated, sterigmata 3–5 µm long, with clamp connections at the bases. ***Cheilocystidia*** 40–45 × 10–15 µm, broadly clavate to clavate, some with acute apices 4–6 µm long. ***Pleurocystidia*** similar to cheilocystidia, lanceolate to clavate, some with sub-acute to sub-capitate apices, appendix longer (6–8 µm) than with cheilocystidia. ***Pileipellis*** a hymeniform layer, consisting of broadly clavate, clavate to fusiform elements, 20–25 × 10–20 µm, strongly encrusted with golden brown pigment. Hyphae of the universal veil thin walled, cylindrical, 3–6 µm in diameter, strongly encrusted with golden brown pigment, clamp connections present.

**Figure 4.**
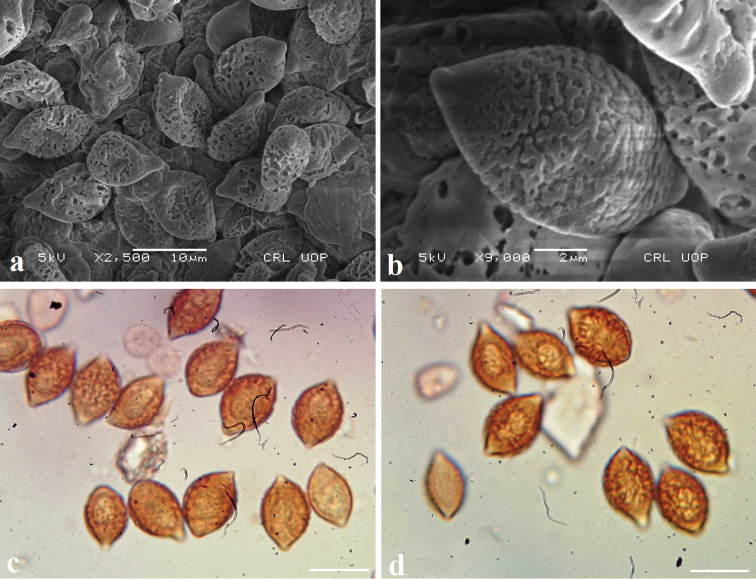
**a–d** Basidiospores of *Descolea quercina* (MJ-1590) **a**, **b** SEM **c**, **d** in KOH solution. Scale bars 10 µm for **a, c, d**; 2 µm for **b**.

#### Known distribution.

PAKISTAN, Khyber Pakhtunkhwa province, Swat district, Malam Jabba valley, Kishawra village. PAKISTAN Khyber Pakhtunkhwa Province, Shangla district, Toa valley.

#### Ecology.

Associated with *Quercus* species. Season July-August

#### Etymology.

The epithet “quercina” refers to association of this taxon with *Quercus* species.

#### Conservation status.

The species is very rare and is currently reported from two locations in the districts of Shangla and Swat in the northern areas of Khyber Pakhtunkhwa province, Pakistan.

#### Additional specimens examined.

Pakistan, Khyber Pakhtunkhwa province, Shangla district, Toa valley, 2000 m alt., among decomposing litter under *Quercus incana*, 15 July 2015, Arooj Naseer, AST33, (LAH35218). Pakistan, Khyber Pakhtunkhwa province, Swat district, Malam Jabba valley, 1900 m alt., on soil under *Quercus dilatata* Royle, 25 July 2015, Junaid Khan, MJ-1590a, (LAH35219).

**Figure 5.**
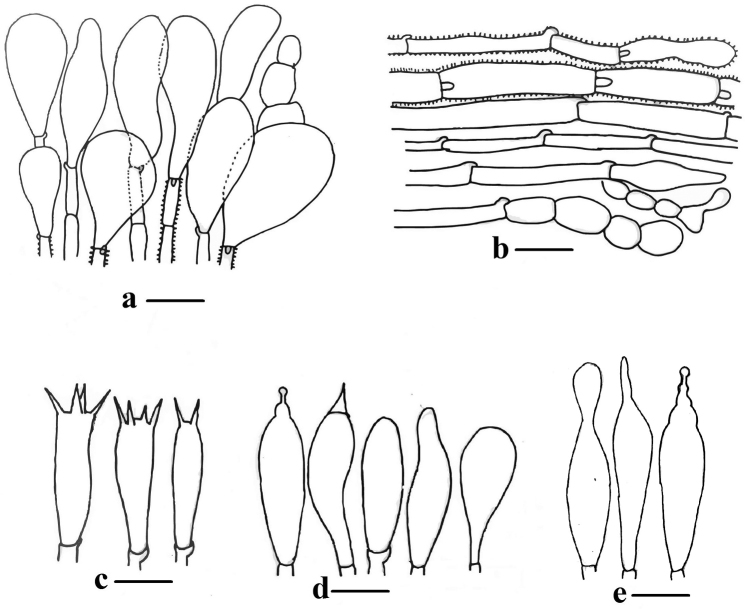
**a–e** Microscopic structures of *Descolea quercina* (Holotype): **a, b** Pileipellis **c** Basidia **d** Cheilocystidia **e** Pleurocystidia. Scale bars 13 µm for **a, b**; 16 µm for **c–e**.

## Discussion

*Descolea quercina* is characterized by medium to large basidiomata, with light yellowish brown to deep yellowish brown color, hygrophanous and squamose to squamose-granulose pileus, light brown to yellowish brown stipe with strongly striated annulus and fibrillose base, and limoniform, coarsely verrucose basidiospores with a smooth apiculus covering the partly concrescent verrucae.

Morphologically, *D. quercina* and *D. pretiosa* E. Horak, resemble each other, in particular because of similar sized basidiomata, color, and limoniform basidiospores. However, *D. pretiosa* differs from *D. quercina* by its strongly scaly pileus, somewhat larger basidiospores (12–14.5 × 7–8 µm) with isolated warts, and habitat under conifers (Horak 1971). *Descolea majestatica* E. Horak, because of similar size, color, and strongly warted basidiospores with a plage could also be misidentified as *D. quercina*. However, *D. majestatica* is easily differentiable by its slimy pileus lacking squamules and larger (12.5–15 × 7–8 µm), amygdaliform basidiospores (Horak 1971). Based on these differences and phylogenetic evidence *D. majestatica* recently was transferred to *Cortinarius*, *C. majestaticus* (E. Horak) T.P. Anderson & Orlovich (Anderson and Orlovich 2016). *Descolea flavoannulata* (Lj.N. Vassiljeva) E. Horak, another large-sized taxon already reported from Pakistan (Niazi et al. 2007), resembles *D. quercina* by somewhat similar basidiomata colors and limoniform basidospores, however, it has a radially wrinkled pileus and larger basidiospores (12–16 × 8–9 µm) without plage.

Based on phylogenetic evidence, *D. quercina* is sister to a clade circumscribing *D. maculata*, *D. gunnii* and *D. recedens*. *Descolea maculata* also has a pileus with appressed squamulae, similar colored basidiomata, and basidiospores of almost the same size (10–13 × 6–7.5 µm). But *D. maculata* has a rippled or wrinkled pileus surface and amygdaliform to sublimoniform basidiospores, which are minutely verrucose (Bougher and Malajczuk 1985). Comparison of *D. quercina* with other closely related species is given in Table 1.

**Table 1.** Morphological comparison of *Descolea quercina* with morphologically similar species.

Characters/ Species	Size of fruiting body	Color	Surface features	Size and shape of basidiospores	Ornamentation	Associated with
*D. quercina* sp. nov.	Pileus 50–70mm, stipe 50–70 × 8–12 mm	Light yellowish brown to deep yellowish brown with or without olivaceous tinge	Squamose to squamose-granulose, margin striated	11.5–13 × 6.7–8.6 µm, Q = 1.5, limoniform	Coarsely verrucose, verrucae partly concrescent	*Quercus*
*D. flavoannulata* (Lj.N. Vassiljeva) E. Horak.	Pileus 50–80 mm, stipe 60–100 × 7–10 mm	Melleous ocher to dark brown	Radially wrinkled, sprinkled with concentrically arranged, small, floccose scales	12–16 × 8–9 µm , limoniform	Coarsely verrucose	*Castanopsis*, *Larix*, *Pinus*, *Quercus*
*D. gunnii* (Berk. ex Massee) E. Horak	Pileus 10–45 mm, stipe 15–60 × 1.5–7 mm	Dark brown to ochraceus	Densely appressed fibrillose-squamulose, striated at the margin,	9.5–12 × 6–7 µm, sub–limoniform	Verrucose with smooth mucro	*Leptospermum*, *Nothofagus*
*D. pallida* E. Horak	Pileus 10–40 mm, stipe 20–60 × 2–5 mm	Yellowish to reddish-brownish	Distinctly slimy, radially wrinkled, striated at the margin	10–13 × 5–6.5 µm, amygdaliform–limoniform	Isolated minute warts	*Nothofagus*
*D. phlebophora* E. Horak	Pileus 10–30 mm, stipe 30–70 × 2–6 mm,	Reddish brown to liver brown	Deeply wrinkled at the center and radially veined, striate near the margin, veil remnants absent	8–11.5 × 5–6 µm, amygdaliform	Minutely warted	*Nothofagus*
*D. pretiosa* E. Horak	Pileus 70–85 mm, stipe 75–80 × 11–13 mm	Fuscous with slight olivaceous tinge to date brown when moist, becoming rich brownish ochraceous with olivaceous tinge	Strongly rugulose, with small, floccose, loose scales	12–14.5 × 7–8 µm, limoniform	Strongly verrucose by isolated warts	*Abies*, *Picea*, *Pinus*, *Taxus*
*Cortinarius majestaticus* (E. Horak) T.P. Andreson & Orlovich	Pileus 30–70 mm, stipe 40–80 × 8–15 mm	Dark brown with a olive-greenish tinge	Slimy, without squamules, margin striated and there wrinkled	12.5–15 × 7–8 µm, amygdaliform	Strongly warted	*Nothofagus*

*Descolea quercina* is a striking new species associated with *Quercus* in temperate areas of Pakistan. The ecology and biogeography of this species are particularly significant since most *Descolea* species associated with Fagaceae are native to the Southern hemisphere (New-Zealand, Australia, South America). The only known *Descolea* species associated with *Quercus* or *Castanopsis* and occurring in the Northern hemisphere are now *D. flavoannulata* and *D. quercina*.

## Supplementary Material

XML Treatment for Descolea quercina
